# Transposon mutagenesis of *Rickettsia felis sca1* confers a distinct phenotype during flea infection

**DOI:** 10.1371/journal.ppat.1011045

**Published:** 2022-12-21

**Authors:** Hanna J. Laukaitis, Triston T. Cooper, Chanakan Suwanbongkot, Victoria I. Verhoeve, Timothy J. Kurtti, Ulrike G. Munderloh, Kevin R. Macaluso

**Affiliations:** 1 Department of Microbiology and Immunology, University of South Alabama College of Medicine, Mobile, Alabama, United States of America; 2 Department of Microbiology and Immunology, University of Maryland School of Medicine, Baltimore, Maryland, United States of America; 3 Department of Entomology, University of Minnesota, St. Paul, Minnesota, United States of America; Washington State University College of Veterinary Medicine, UNITED STATES

## Abstract

Since its recognition in 1994 as the causative agent of human flea-borne spotted fever, *Rickettsia felis*, has been detected worldwide in over 40 different arthropod species. The cat flea, *Ctenocephalides felis*, is a well-described biological vector of *R*. *felis*. Unique to insect-borne rickettsiae, *R*. *felis* can employ multiple routes of infection including inoculation via salivary secretions and potentially infectious flea feces into the skin of vertebrate hosts. Yet, little is known of the molecular interactions governing flea infection and subsequent transmission of *R*. *felis*. While the obligate intracellular nature of rickettsiae has hampered the function of large-scale mutagenesis strategies, studies have shown the efficiency of *mariner*-based transposon systems in Rickettsiales. Thus, this study aimed to assess *R*. *felis* genetic mutants in a flea transmission model to elucidate genes involved in vector infection. A *Himar1* transposase was used to generate *R*. *felis* transformants, in which subsequent genome sequencing revealed a transposon insertion near the 3’ end of *sca1*. Alterations in *sca1* expression resulted in unique infection phenotypes. While the *R*. *felis sca1*::*tn* mutant portrayed enhanced growth kinetics compared to *R*. *felis* wild-type during *in vitro* culture, rickettsial loads were significantly reduced during flea infection. As a consequence of decreased rickettsial loads within infected donor fleas, *R*. *felis sca1*::*tn* exhibited limited transmission potential. Thus, the use of a biologically relevant model provides evidence of a defective phenotype associated with *R*. *felis sca1*::*tn* during flea infection.

## Author summary

First discovered in the early 1990’s, *Rickettsia felis* has now been identified in arthropod vectors across the globe. The bacteria are primarily transmitted by cat fleas through horizontal and vertical transmission routes. While transmission biology is being defined for *R*. *felis*, the genetic factors facilitating rickettsial infection and subsequent transmission are unknown. Understanding rickettsial requirements to infect their vector hosts is paramount to developing intervention approaches. Here, we generated novel *R*. *felis* genetic mutants to uncover potential genes involved in crucial steps of flea infection and transmission. We identified that a mutation in surface cell antigen-1 (*sca1*) resulted in limited ability of rickettsiae to attach to arthropod cells *in vitro*. Additionally, we observed a decrease in rickettsial loads of the mutant strain during flea infection when compared to the wild-type strain, suggesting that *sca1*-mediated attachment to midgut cells (a primary step after acquisition) is crucial in establishing infection within the flea. Our study provides a platform and tools to further examine the interrelationship between *Rickettsia* and their arthropod hosts.

## Introduction

Rickettsial pathogens are obligate intracellular bacteria spread by hematophagous arthropods associated with a spectrum of emerging and reemerging vector-borne diseases worldwide. In the United States, there has been a resurgence of flea-borne rickettsioses within endemic areas, including California, Texas, and Hawaii [1]. Among flea-borne rickettsiae, *Rickettsia felis*, the causative agent of flea-borne spotted fever (FBSF), has been detected worldwide in over 40 different arthropod species [2,3]. Since being associated with human infection in 1994 [4], further incriminating evidence implicating *R*. *felis* as a widely distributed human pathogen is building. As a common cause of febrile illness in sub-Saharan Africa, FBSF is found to be prevalent among 3–15% of hospitalized patients diagnosed with fevers of unknown origin, but underestimation of the perceived risk is likely due to shared similarities in clinical signs (fever, headache, myalgia) with other endemic febrile illnesses [3,5]. Moreover, a recent study demonstrates successful transmission from *R*. *felis-*infected fleas to canine hosts, resulting in a rickettsemic infection [6]. The cat flea, *Ctenocephalides felis*, which is a predominant ectoparasite found on domestic and wild animals, is a well-described biological vector of *R*. *felis* [7,8]. Notably, *R*. *felis* can utilize multiple routes to infect vertebrate hosts, including inoculation of infectious salivary secretions and potentially infectious flea feces [9–13]. While recognized as an emerging pathogen, little is known of the molecular interactions governing flea infection and subsequent transmission of *R*. *felis*.

Genetic modification of vector-borne pathogens, such as *Yersinia pestis* and *Bartonella henselae*, has identified bacteria-derived factors essential for infection or transmission in fleas [14–20]. In contrast to these extracellular pathogens, the fastidious nature of rickettsiae requires direct interaction with host cells for propagation, complicating the development of applicable molecular tools. Utilizing genetic manipulation, studies have implicated several rickettsial determinants, including surface cell antigen-0 (Sca0), Sca1, Sca2, Sca4, Sca5, RickA, and RalF, in adhesion, invasion, cell-to-cell spread, and/or avoidance of the immune response in a mammalian host system [21–26]. While rickettsial factors vital for infection are being elucidated in vertebrates, less is known in arthropod vectors. For example, Sca1 is known to be expressed on the surface of rickettsiae and facilitate attachment to non-phagocytic mammalian cells during *in vitro* culture [21]; however, its role during vector infection remains unknown.

Arthropod-borne pathogens undergo complex changes in their host environment as they traverse between vector and vertebrate hosts. It is known that *R*. *felis* utilizes host-specific gene regulation during infection and transmission by the arthropod vector [27]. Likewise, fleas mount an immune response against invading rickettsiae [13,28,29]. The kinetics of *R*. *felis* infection in the flea has been detailed, with rickettsiae observed throughout the midgut, excretory system, salivary glands, and ovarian tissues as early as 7 days post-exposure, indicating mechanisms of immune evasion have evolved [30,31]. However, the rickettsial determinants driving flea infection remain to be elucidated. Therefore, the objective of this study was to characterize the phenotype of a *R*. *felis* transformant during flea infection. The establishment of an intracellular niche is crucial for rickettsial survival; therefore, it is hypothesized that if *sca1* is essential in the vector, then disruption will result in an altered infection phenotype. In the current study, a *R*. *felis sca1*::*tn* mutant was generated and utilized in an arthropod host system to determine its contribution to infection and transmission. While the *R*. *felis sca1*::*tn* mutant portrayed enhanced growth kinetics compared to *R*. *felis* wild-type during *in vitro* culture, rickettsial loads were significantly reduced during flea infection. Therefore, the use of a biologically relevant model implicates *sca1* as an essential factor facilitating *R*. *felis* infection in the flea.

## Results

### *Himar1* transposon mutants

**Fig 1:**
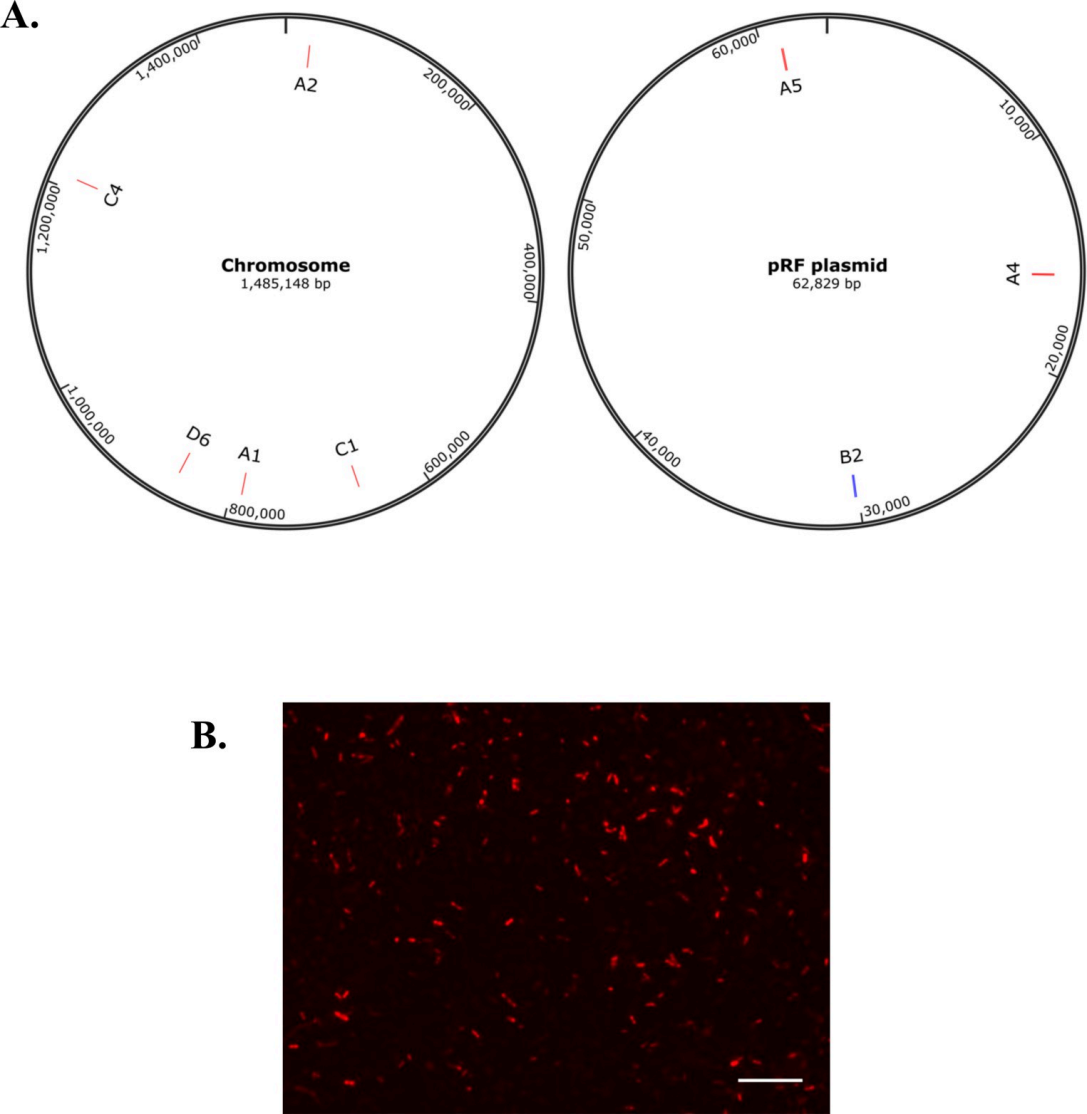
Transposon mutagenesis of *R*. *felis* transformants. A) Chromosomal and pRF plasmid maps indicating *Himar1* insertion sites for each mutant population into coding (red) and intergenic (blue) regions. B) mCherry expression using fluorescence microscopy of sucrose purified A5 mutant grown in ISE6 cells. Scale bar = 50 μm.

**Fig 2:**
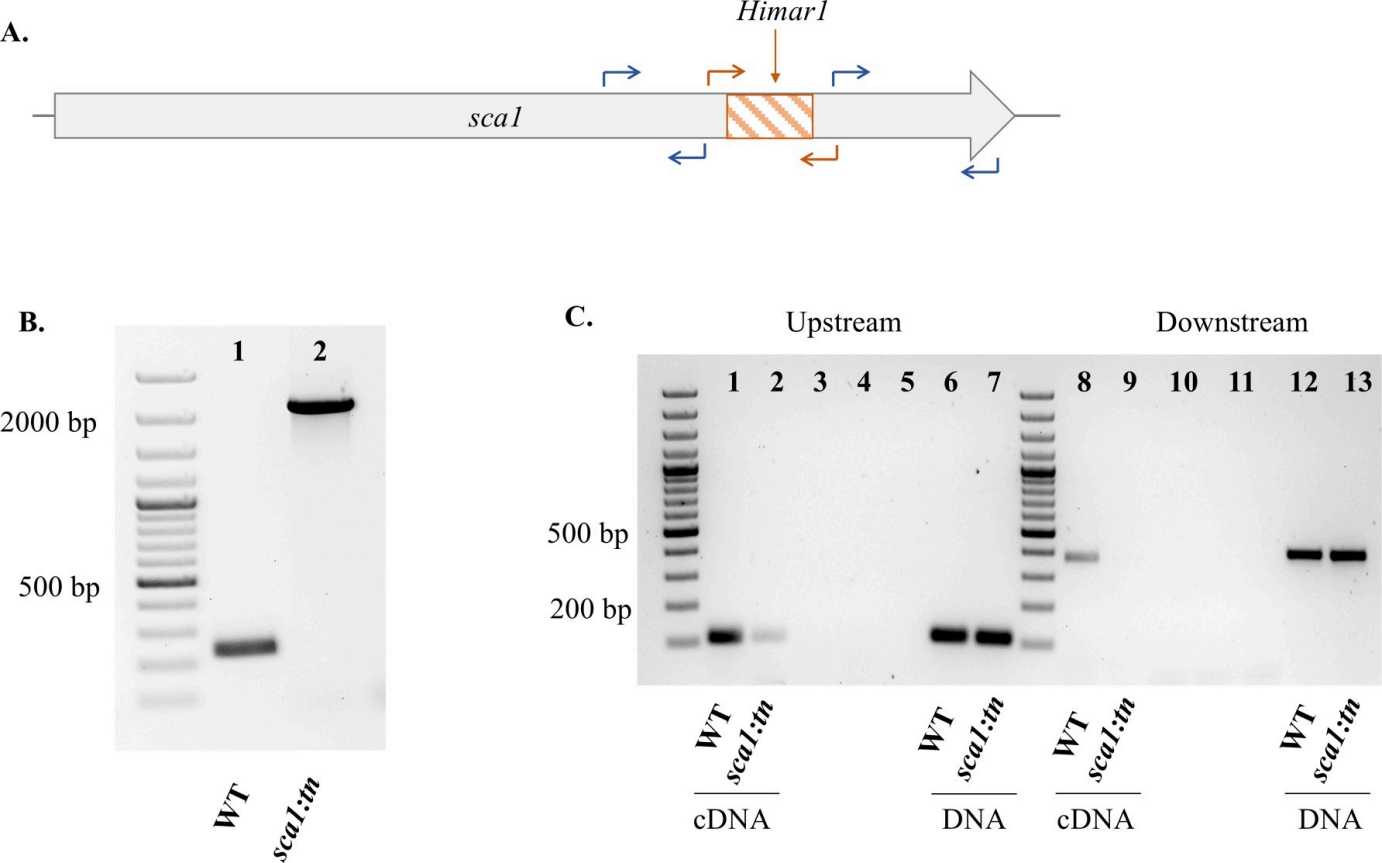
Characterization of *R*. *felis sca1*::*tn* after *Himar1* insertion. A) Graphical representation of the position of *Himar1* insertion (orange) within the *R*. *felis* genome. Annotated with primer sets for clonality (orange arrows) and RT-PCR (blue arrows). B) Agarose gel depicting clonality *of R*. *felis sca1*::*tn* (lane 2) using primers flanking the insertion site and *R*. *felis* WT (lane 1) as a control. C) Using RT-PCR, the transcriptional profile of *sca1* in *R*. *felis* WT (lane 1 and 8) and *R*. *felis sca1*::*tn* (lane 2 and 9) upstream and downstream of the *Himar1* insertion site, respectively. Rickettsial DNA samples were used as controls for gene amplification (lanes 6, 7, 13, 14). cDNA samples lacking reverse transcriptase were used as a negative control (3, 4, 10, 11). PCR reaction without template is shown in lane 5.

Using a modified pCis-mCherry-SS *Himar* A7 plasmid [32], rickettsial mutants were generated from *R*. *felis* str. LSU. Whole genome sequencing identified *Himar1* insertion sites for 5 mutants, with the remaining 3 detected by semi-random nested PCR alone. Sequencing results were confirmed by PCR amplification, cloning, and Sanger sequencing. Results indicate 8 insertion sites with representatives in both *R*. *felis* chromosomal DNA, as well as the pRF plasmid (**Fig 1A** and **Table 1**). By microscopy, mCherry fluorescence was observed for some *R*. *felis* transformants (**Fig 1B**). To achieve clonal populations, the limiting dilution method was used and *R*. *felis* mutants were grown under selective culture in ISE6 cells using L15B medium supplemented with spectinomycin and streptomycin [33]. For further characterization of growth phenotypes, a single mutant, *R*. *felis sca1*::*tn*, was selected. Sanger sequencing results indicated *Himar1* insertion near the 3’ end of *sca1* (**Fig 2A**). Clonality was confirmed by PCR amplification of the flanking regions of the transposon (**Fig 2B**).

**Table 1:** Transposon insertion sites for *R*. *felis* mutants.

Mutant	Accession	Genomic insertion site	Direction	Locus ID	Gene product description
A1	SAMN31560795	788,263	anti-sense	RF_0725	Surface cell antigen 4
A2	SAMN31560790	25,261	sense	RF_0022	Surface cell antigen 1
A4	SAMN31560792	15,820	sense	RF_p18	Tetratricopeptide repeat domain protein
A5	SAMN31560794	60,888	anti-sense	RF_p66	Site-specific recombinase (tail end)
B2	SAMN31560793	30,129	n/a	Intergenic region of pRF plasmid	n/a
C1	SAMN31560791	664,857	sense	RF_0622	Unknown protein
C4	N/A	1,211,812	sense	Rf_1144	Unknown protein
D6	N/A	857,160	anti-sense	RF_0808	5-Formyltetrahydrofolate cyclo-ligase

### *Himar1* transposon interrupts normal gene transcription of *sca1*

To assess the effect of the transposon insertion on *sca1* gene transcription, total RNA was isolated from *R*. *felis sca1*::*tn* and *R*. *felis* WT and analyzed by reverse transcriptase PCR (RT-PCR). Primers designed to amplify upstream of the *Himar1* insertion site showed a reduction in *sca1* expression in *R*. *felis sca1*::*tn* compared to *R*. *felis* WT (**Fig 2C**). Furthermore, amplification of the downstream region of *sca1* revealed an ablated gene expression for *R*. *felis sca1*::*tn* (**Fig 2C**). This data suggests that the transposon had an impact on normal *sca1* mRNA synthesis at the 3’ end of the gene.

As it is known that transposon insertions can have polar effects on adjacent genes [32,34,35], primers were designed to amplify cDNA of portions of 3 genes located downstream of *sca1*, *RF_0023*, *RF_0024*, and *RF_0025* (**S1A Fig**). Of interest, only transcripts from *RF_0023* were detected in *R*. *felis* WT, where the absence of transcripts was observed for *R*. *felis sca1*::*tn* (**S1B Fig**). No amplification of rickettsial DNA was detected in no reverse transcriptase controls. Thus, the data suggests the *Himar1* insertion has altered *sca1* gene transcription and subsequently affected transcription of a neighboring gene.

### *R*. *felis sca1*::*tn* portrays deficiency in tick cell attachment

**Fig 3:**
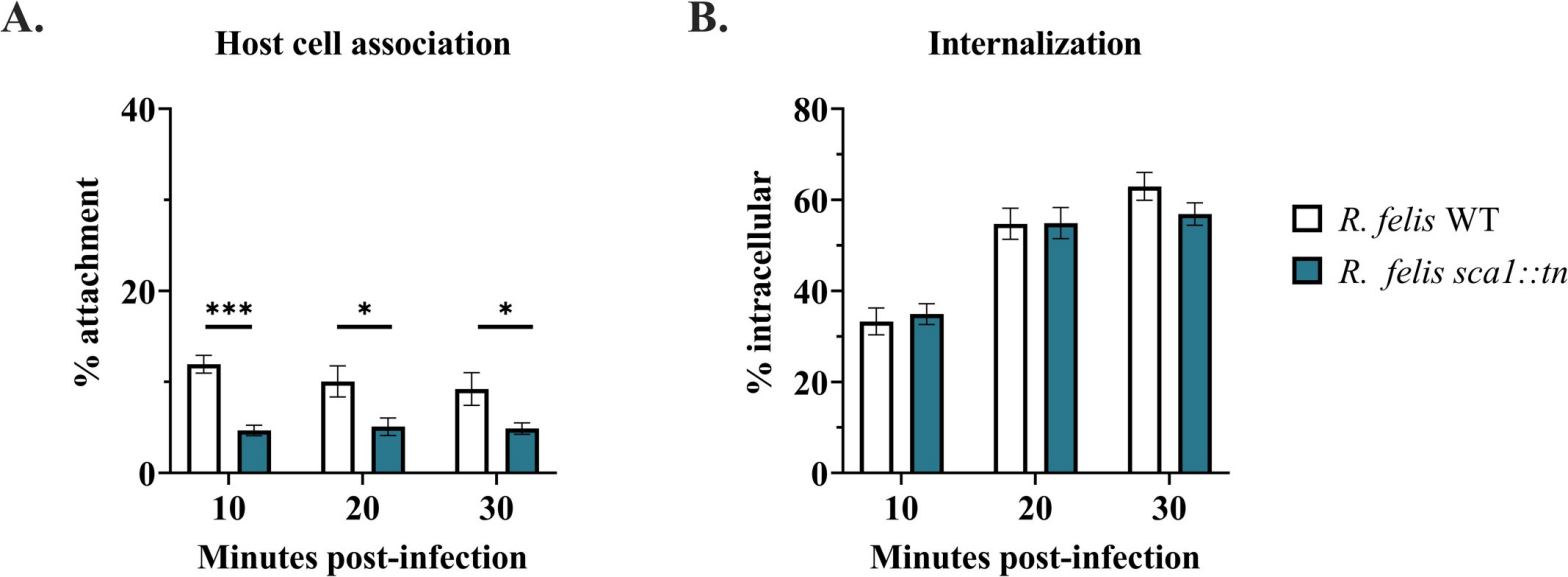
Cell association and invasion. Semi-purified rickettsial strains were infected onto ISE6 cells at a MOI of 10. A) Percent of *R*. *felis* WT (white) or *R*. *felis sca1*::*tn* (teal) attached to host cells was calculated as a ratio of extracellular rickettsiae per host cell nuclei. B) Percent of internalized *R*. *felis* WT (white) and *R*. *felis sca1*::*tn* (teal) was quantified in relation to total rickettsiae (intracellular/total rickettsiae). Data are representative of means ± SEM from 2 independent replicates for a total of 20 fields of view. Significance was assessed by unpaired t-test (* *p*<0.05; ** *p*<0.01); *** *p*<0.001) indicating the mean varies significantly from wild-type at a given time point.

Sca1 has previously been shown to play a role during adhesion to a non-phagocytic mammalian cell line [21]. To determine if a similar phenotype occurs for *R*. *felis* during arthropod cell infection, the number of host cell-associated rickettsiae were quantified by microscopy and compared between *R*. *felis* WT and *R*. *felis sca1*::*tn* within the first 30 minutes of infection. The results identify a significant decrease in the ability of the *R*. *felis sca1*::*tn* mutant to attach to host cells across all time points examined (**Fig 3A**). However, the *R*. *felis* mutant was internalized to a similar degree to that of *R*. *felis* WT (**Fig 3B**). As previously described for the role of *R*. *conorii* Sca1 [21], the results presented are consistent with established redundant mechanisms of cell entry and suggest an independent mechanism occurs between attachment and invasion.

### Enhanced growth of *R*. *felis sca1*::*tn* in tick cells

**Fig 4:**
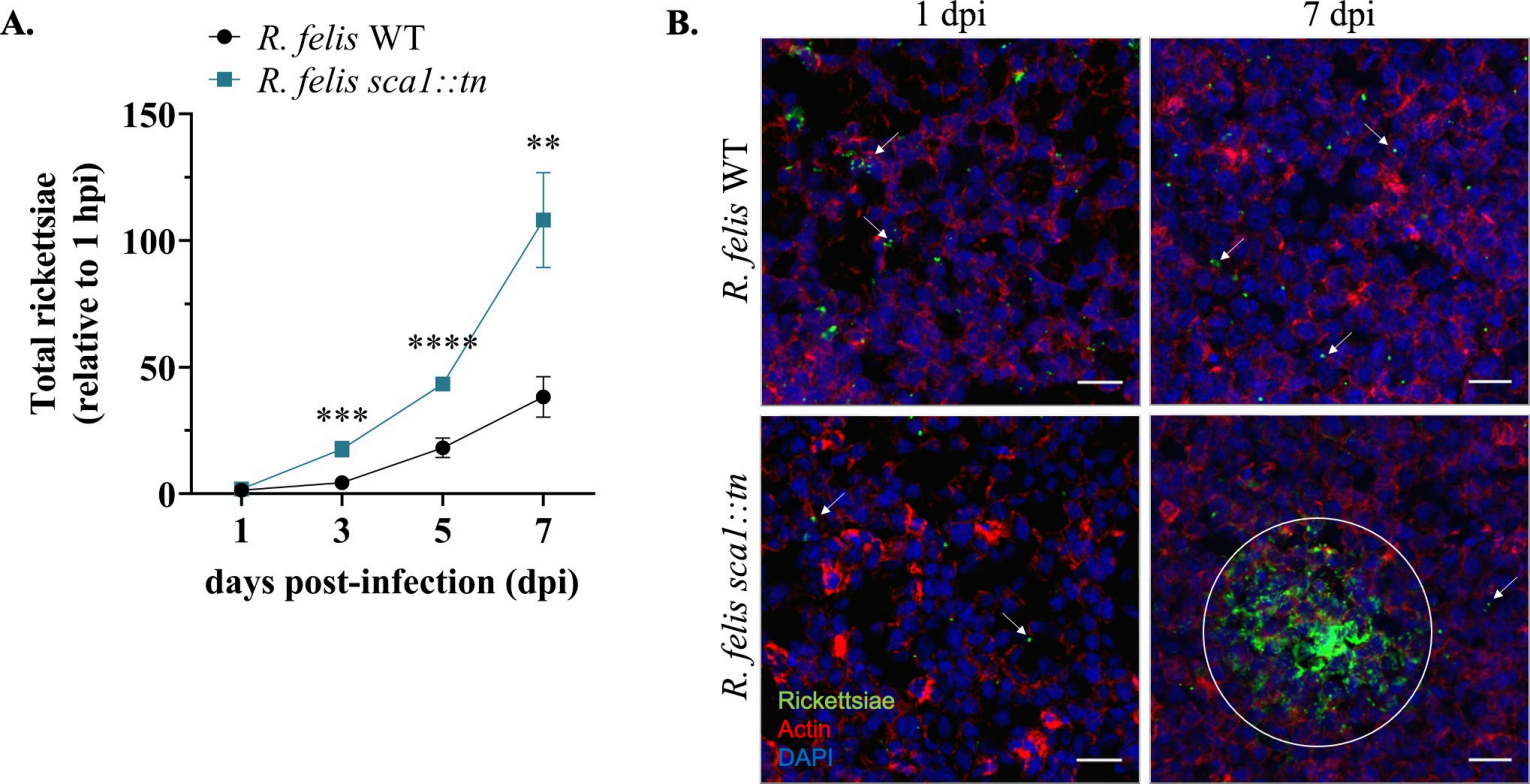
Growth kinetics of *R*. *felis sca1*::*tn* in tick cells. A) Growth curves of *R*. *felis* WT (black) and *R*. *felis sca1*::*tn* (teal) in ISE6 cells, measuring rickettsial genome equivalents by qPCR. Data is normalized to input bacteria at 1 hpi and representative of the mean ± SEM from three experiments with 3 technical replicates. Significance was assessed by unpaired t-test (** *p*<0.01); ***p<0.001; ****p<0.0001) indicating the mean varies significantly from wild-type at a given time point. B) Fluorescent microscopy images of *R*. *felis* WT and *R*. *felis sca1*::*tn* (green), host cell nuclei (blue), and host actin (red). Arrows indicate rickettsiae. Circle represents dense infection foci of *R*. *felis sca1*::*tn*. Scale bar = 10 μm.

To assess the *R*. *felis sca1*::*tn* mutant’s growth kinetics during arthropod cell culture, both rickettsial strains were independently cultivated in ISE6 cells and genomic equivalents were quantified temporally over a 7-day period by qPCR. Although *R*. *felis sca1*::*tn* was altered in its ability to attach to host cells, its growth was significantly enhanced beginning at 3 dpi when compared to *R*. *felis* WT (**Fig 4A**). Additionally, upon microscopy analysis, *R*. *felis sca1*::*tn* displayed distinct dense foci of infection, whereas *R*. *felis* WT presents a disseminated state of infection with few rickettsiae per cell at 7 dpi (**Fig 4B**) Overall, temporal examination of the growth kinetics following *sca1* mutation indicates enhanced infection within tick cells with a unique phenotype resulting in reduced dissemination.

### *R*. *felis sca1*::*tn* demonstrates a deficient growth phenotype during flea infection

**Fig 5:**
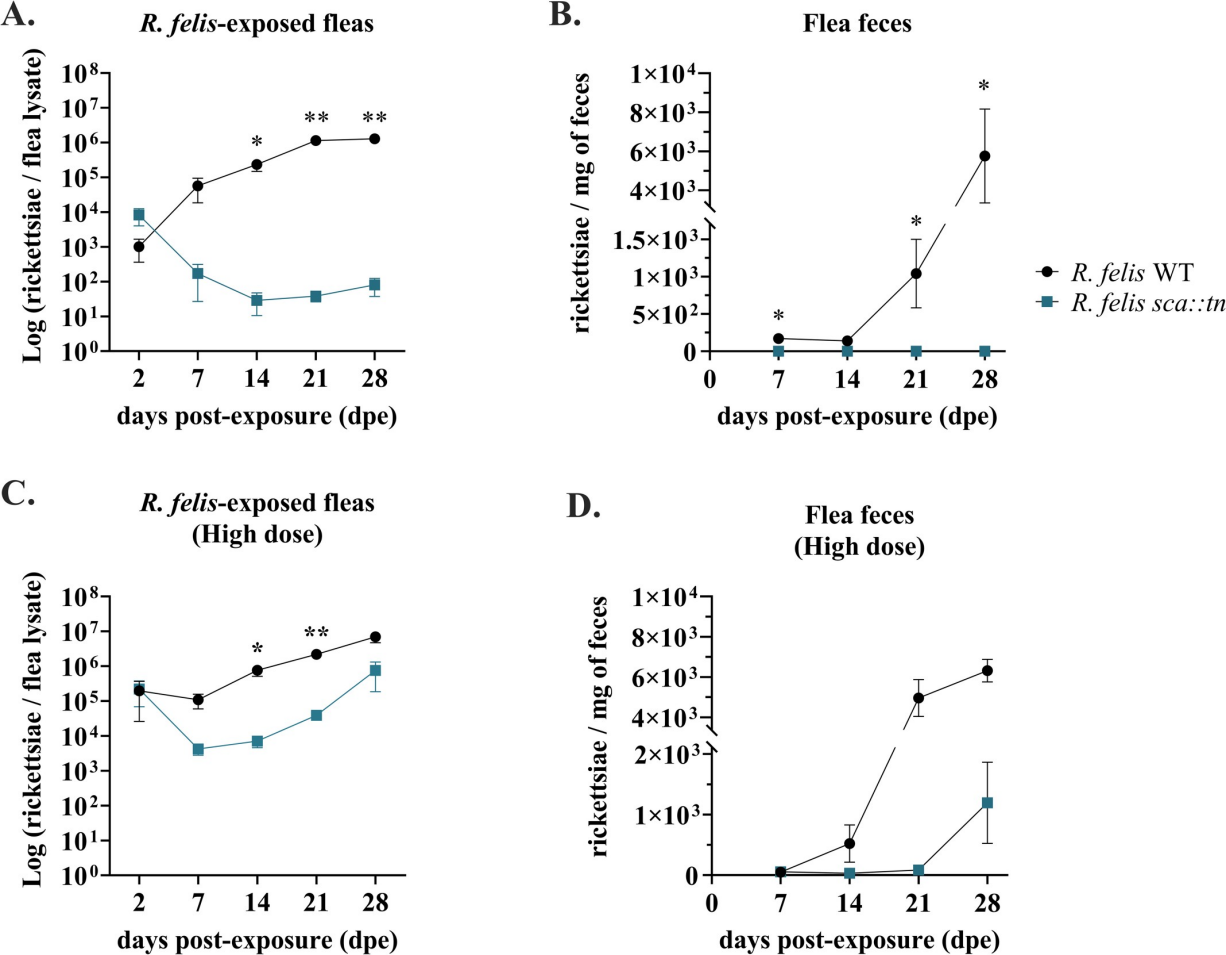
Rickettsial loads during flea infection. A) Fleas were exposed, independently, to *R*. *felis* WT- (black) or *R*. *felis sca1*::*tn*-infected bloodmeals (teal) at an infectious dose of 1.5 x 10^10^ rickettsiae for 48 hours. Data are representative of mean ± SEM from three experiments for a total of 60 fleas with 3 technical replicates. B) Feces collected from exposed fleas were assessed for rickettsial enumeration by qPCR and standardized to 1 mg of feces. C) Fleas were exposed, independently, to *R*. *felis* WT- (black) or *R*. *felis sca1*::*tn*-infected bloodmeals (teal) at an infectious dose of 5 x 10^10^ rickettsiae for 48 hours. Data are representative of mean ± SEM from two experiments for a total of 20 fleas with 3 technical replicates. D) Rickettsial loads per 1 mg of feces collected from fleas exposed to a high dose of rickettsiae. Significance was assessed at a 95% confidence interval (* *p*<0.05; ** *p*<0.01) by two-way ANOVA with Bonferroni’s multiple-comparison test to assess variation in the means from wild-type over time.

**Fig 6:**
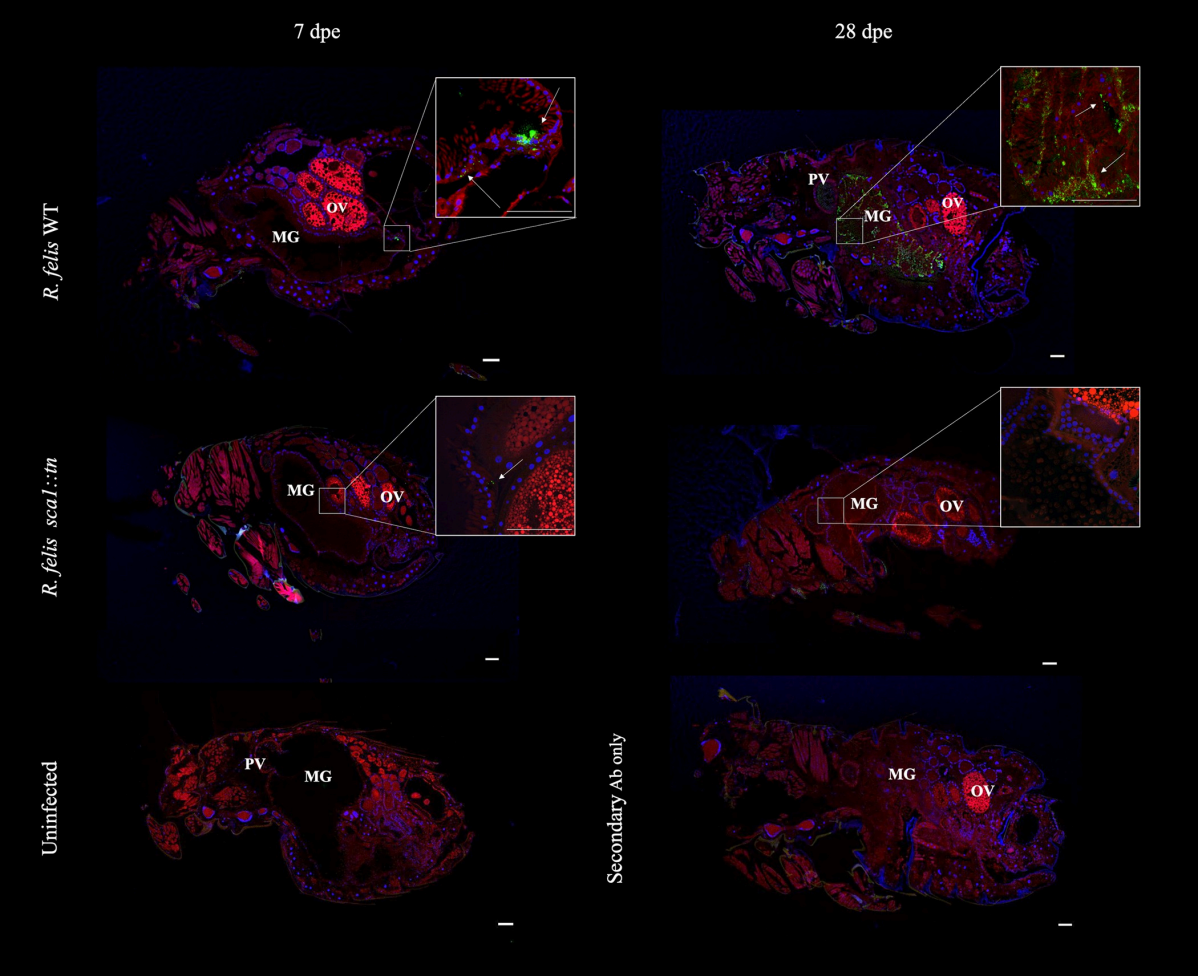
Rickettsial dissemination within fleas. Fleas were exposed to a *R*. *felis* WT- (top panels) or *R*. *felis sca1*::*tn*-infected (middle panels) bloodmeal for 48 hours and female flea sections were stained for rickettsiae (green), nuclei (blue), and Evans blue (red) at 7 dpe (left panels) and 28 dpe (right panels). White arrows indicate rickettsiae. An uninfected flea (bottom left panel) and control lacking primary antibody (bottom right panel) served as negative controls. Image is representative of 4 sectioned fleas at each time point from independently exposed flea cohorts. MG: midgut, PV: proventriculus, OV: ovaries. Scale bar = 100 μm.

To confirm there was not a loss in fitness of the mutant during bloodmeal acquisition assays, rickettsiae were re-isolated from blood following a 48-hour incubation period. Although overall rickettsial growth kinetics were reduced after re-isolation from blood, rickettsiae remained infectious. Specifically, there is no indication that there was statistically significantly reduced *R*. *felis sca1*::*tn* growth compared to *R*. *felis* WT (**S2 Fig**), suggesting the resulting flea infection phenotype is due to factors encountered following bloodmeal ingestion. Due to the inability to generate a rickettsemic model under laboratory settings, the rickettsemia levels in infected hosts remains unknown. To assess the mutant’s phenotype during flea infection, cat fleas were exposed to an absolute number of 1.5 x 10^**10**^ *R*. *felis sca1*::*tn* or *R*. *felis* WT in 600 μl of blood for 48 hours, providing a *Rickettsia*-rich meal and a higher probability of rickettsial infection among feeding fleas [11–13,31]. Following exposure, a subset of fleas was assessed for rickettsial burden, revealing that both flea cohorts acquired comparable loads of rickettsiae. However, weekly assessments over a 28-day period identified significantly lower *R*. *felis sca1*::*tn* loads in individual fleas when compared to *R*. *felis* WT (**Fig 5A**). Additionally, *R*. *felis* WT was detected at increasing levels in flea feces over time, with the highest loads at 28 dpe (**Fig 5B**). Failure of *R*. *felis sca1*::*tn* to replicate to comparable levels as *R*. *felis* WT coincided with the lack of detection in flea feces. However, detection of *R*. *felis sca1*::*tn* in flea feces could be induced, as fleas exposed to a higher dose of rickettsiae (5 x 10^**10**^ total rickettsiae) sustained higher levels of *R*. *felis sca1*::*tn* over time (**Fig 5C and 5D**). Additionally, *R*. *felis sca1*::*tn* was observed at lower rickettsial densities when compared to *R*. *felis* WT by microscopy of whole flea sections, supporting the qPCR results (**Fig 6**). As comparable loads of both strains were detected in fleas at the time of acquisition, the data suggest that *R*. *felis sca1*::*tn* had a deficiency in initiating early flea infection. However, detection of *R*. *felis sca1*::*tn* 28-days after exposure, implies persistence over time within the flea vector.

### *R*. *felis sca1*::*tn* is transmissible during flea cofeeding

**Fig 7:**
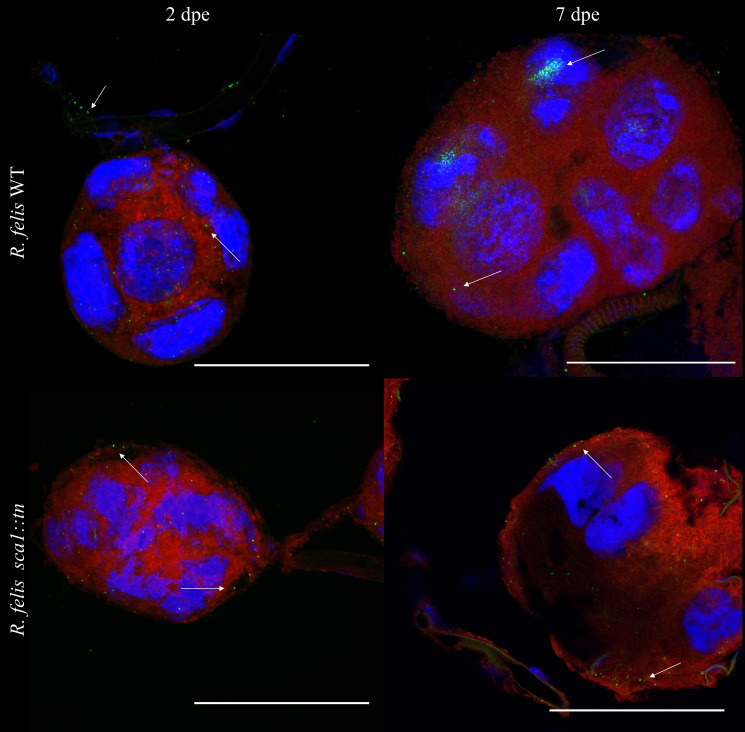
Rickettsial detection within flea salivary glands. Fleas were exposed to a *R*. *felis* WT- (top panels) or *R*. *felis sca1*::*tn*-infected (bottom panels) bloodmeal for 48 hours, in which female salivary glands were micro dissected at 2 dpe and 7 dpe. Samples were stained for rickettsiae (green), nuclei (blue), and Evans blue (red). White arrows indicate rickettsiae. Image is representative of salivary glands dissected from 5 fleas collected from independently exposed flea cohorts. Scale bar = 100 μm.

**Fig 8:**
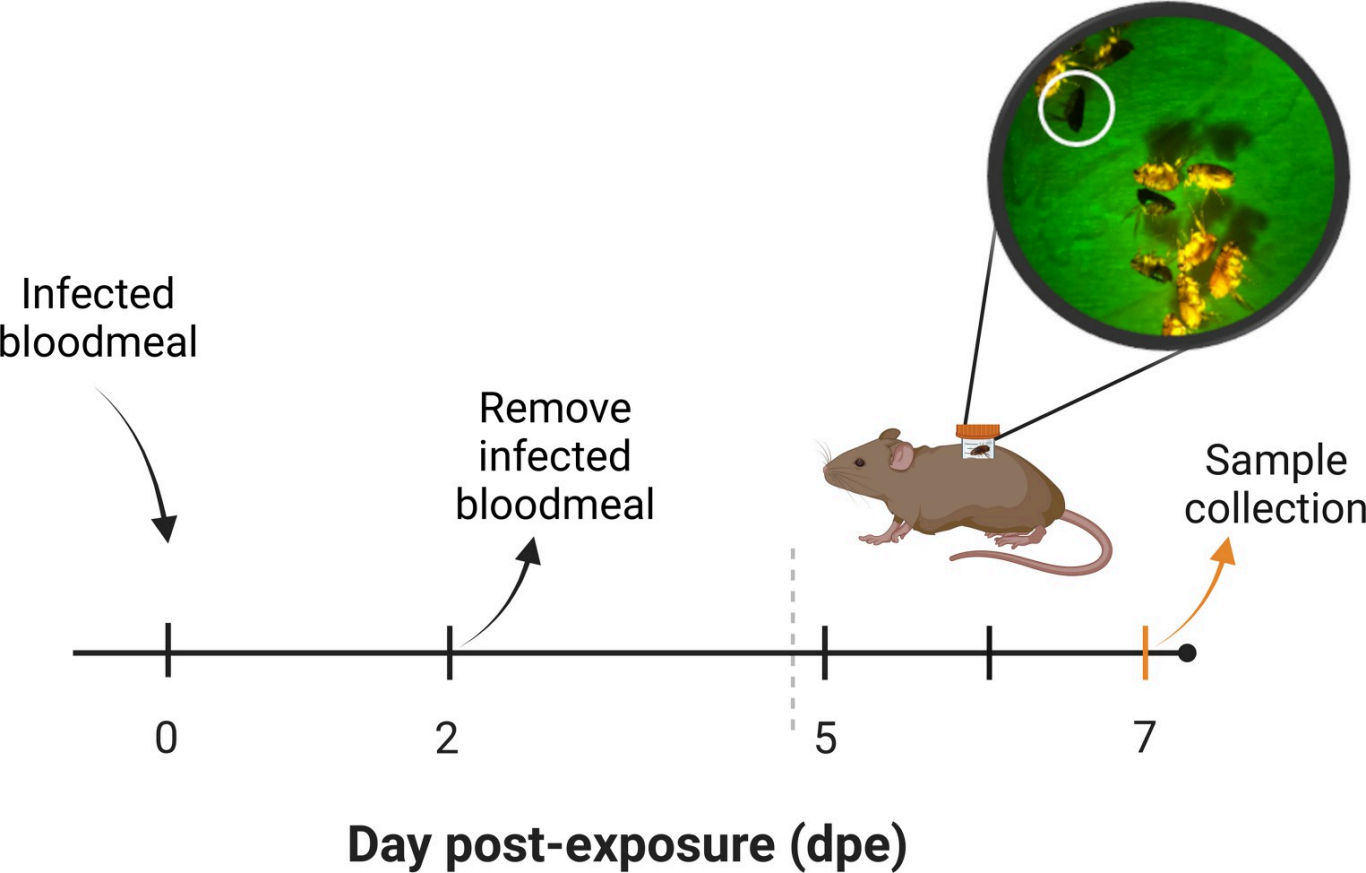
Experimental design for flea cofeeding bioassay. Fleas were independently exposed to a *R*. *felis* WT- or *R*. *felis sca1*::*tn*-infected bloodmeal for 48 hours. At 5 dpe,10 donor (circled) and 10 recipient (yellow) fleas were allowed to feed on a murine host for 12-hour increments, totaling 36 hours. Post-feeding, fleas were individually assessed for rickettsiae by qPCR. Figure created with BioRender.com.

To determine if *R*. *felis sca1*::*tn* disseminated to flea tissues necessary for transmission, such as the salivary glands, fluorescent microscopy was employed. Both rickettsial strains were observed in salivary glands that were recovered from exposed female fleas within 48 hpe and at 7 dpe (**Fig 7**). Detection of the *R*. *felis sca1*::*tn* mutant in the salivary glands of exposed fleas, warranted further investigation into its ability to be transmitted by fleas while feeding on a murine host. Subsequently, a cofeeding bioassay was employed as a means of tracking transmission to proximal feeding arthropods in the presence of a vertebrate host [10,36]. Donor (infected) fleas were allowed to feed with recipient (naïve) fleas for 3 days. Naïve fleas were labeled with the fluorescent biomarker, RhoB, to allow for distinction between the flea cohorts using microscopy (**Fig 8**). Although not significantly different, the cofeeding bioassay generated *R*. *felis* WT infection in 20% of recipient fleas, whereas only 10% of recipient fleas were positive for *R*. *felis sca1*::*tn* by qPCR (**Table 2**). Additionally, rickettsial DNA was identified in the skin of two *R*. *felis* WT-exposed mice and a single *R*. *felis sca1*::*tn*-exposed mouse (**Table 2**), suggesting a mechanism of transmission to naïve fleas. Confirmation of *R*. *felis* in the mouse skin was further validated by Sanger sequencing of PCR-amplified *R*. *felis ompB*. Although comparable prevalence of infected donor fleas (77% and 80%) for *R*. *felis* WT and *R*. *felis sca1*::*tn*, respectively, was observed, rickettsial loads were significantly lower in the *R*. *felis sca1*::*tn*-exposed fleas (**Table 2**). Thus, the data suggests that the *R*. *felis sca1*:*tn* mutant has a decreased capacity to infect fleas, ultimately lowering its transmission potential.

**Table 2:** Rickettsial transmission between cofeeding fleas.

Species	Mean infection loads (± SEM) ****	Donor prevalence (%)	Recipient prevalence (%)	Positive skin samples
*R*. *felis* WT	1.93x10^7^ (± 1.86x10^7^)	23/30 (77)	6/30 (20)	2/3
*R*. *felis sca1*::*tn*	2.10x10^3^ (± 6.08x10^2^)	24/30 (80)	3/30 (10)	1/3

Significance was assessed at the 95% confidence level for differences between rickettsial loads in donor fleas by Mann Whitney test (**** *p*<0.0001).

## Discussion

The genetic manipulation of rickettsiae has historically been challenging. The advent of transposon mutagenesis has allowed for the discovery of rickettsial-specific virulence factors. Phenotypes associated with genetic mutants observed during *in vitro* culture have elucidated functionality [22–24,37,38] or their crucial involvement in causing disease *in vivo* [24,38,39]. Although informative, these studies have primarily occurred in tick associated SFG *Rickettsia* spp. However, distinct genetic and biological differences between tick- and insect-borne rickettsiae exist (reviewed in [40–42]); therefore, the role of these molecules cannot be generalized across the entire *Rickettsia* genus. Moreover, most molecules have been characterized in a mammalian host background, leaving rickettsial biology during arthropod infection comparatively understudied. Our results demonstrate the use of transposon mutagenesis of an insect-borne rickettsiae, *R*. *felis*, to characterize a mutant phenotype in a biologically relevant flea model.

Transposon mutants have provided a platform to study the role of virulence factors during infection in numerous bacterial species, including *Rickettsia* spp. [37,43,44]. As sequencing revealed, the *Himar1* transposon had inserted near the 3’ end of *sca1*. Validation of a clonal, isogenic mutant led to examination of the insertional effects of the transposon in the mutant. For *R*. *felis sca1*::*tn*, reduction of *sca1* transcript was observed both upstream and downstream of the insertion site, suggesting altered mRNA synthesis of the region necessary for translation of the β-peptide domain of the Sca1 protein.

As the Scas belong to a class of immunodominant outer membrane proteins (Omps) that are known to be involved in recognition of, attachment to, and protrusion through host cells [21,26,45,46], they are highly conserved among *Rickettsia* spp., with a core 5 *sca* genes encoded in most rickettsial genomes [47, 48]. Scas are characterized as type-V secretion systems, or autotransporters, which is defined by the presence of (1) an N-terminal signal sequence; (2) a central passenger domain; (3) a C-terminal β-peptide domain [47,49]. As Gram-negative bacteria, the rickettsial membrane consists of an inner membrane (IM), periplasmic space (PP), and outer membrane (OM). Thus, to achieve implantation into the OM, proteins must be secreted across the IM. In bacterial species, such as *Escherichia coli*, the structural integrity of the β-peptide domain is essential in the recognition process during translocation and OM anchoring (reviewed in [47]). The observation of several Scas anchored to the surface of rickettsiae [21,23,24] indicates the essential proteins are present to achieve translocation from the cytosol to the OM. Importantly, the β-peptide domain of another dominant Omp has been associated with cell surface expression and direct host interactions [50]. However, to study the exact function of the β-peptide domain and its role in translocation of the Sca1 peptide to the surface of *R*. *felis*, the *R*. *felis sca1*::*tn* mutant will require further investigation at the protein level. Protein expression was not examined in the current study due to the lack of an available antibody specific to *R*. *felis* Sca1.

Transposons are known to have polar effects on the expression of neighboring genes [32,34]. To determine whether this occurred in *R*. *felis sca1*::*tn*, transcripts from three genes downstream of *sca1* were analyzed. The mutant exhibited a loss of gene expression for *RF_0023* when compared to *R*. *felis* WT. However, genes *RF_0024* and *RF_0025* were undetectable by RT-PCR in either *R*. *felis* WT or *R*. *felis sca1*::*tn* during tick cell culture. The results suggest that while *RF_0024* and *RF_0025* may not be expressed by *R*. *felis* during tick cell infection, the introduction of the transposon in the mutant induced polar effects on an adjacent gene. Due to the lack of comprehensive gene annotation, the influence of *RF_0023* on the *R*. *felis sca1*::*tn* mutant’s phenotype during infection cannot be assigned. However, the ability of the *R*. *felis sca1*::*tn* mutant to infect cells under these experimental conditions suggests that the gene is dispensable for tick cell infection. Future studies detailing transcript activity during *R*. *felis* infection in arthropod cells would provide additional insight into currently uncharacterized genes.

While annotated in all validated *Rickettsia* spp., little is known of the functional role of Sca1 during infection and transmission. Using a non-phagocytic mammalian cell model, pretreatment with Sca1 antibodies reduced the attachment efficiency of *R*. *conorii* to host cells [21]. Due to the current lack of an available flea-derived cell line, a surrogate *in vitro* arthropod system was utilized. ISE6 cells have been routinely adopted to isolate and grow *R*. *felis* isolates [13,51,52]. In the current study, the *R*. *felis sca1*::*tn* mutant was limited in the ability to attach to tick cells, suggesting a universal role of Sca1 across both vertebrate and arthropod hosts. Genetic mutagenesis of *Rickettsia* spp. has revealed unique phenotypes during *in vitro* culture; yet these mutants remain competent in overall growth when compared to wild-type strains [22–24,53,54]. In this study, the *R*. *felis sca1*::*tn* mutant exhibited enhanced growth in tick cells compared to *R*. *felis* WT. The phenotype is consistent with tick-borne rickettsiae, *Rickettsia parkeri sca2*::*tn* and *R*. *parkeri rickA*::*tn*, where altered phenotypes were not observed during *in vitro* culture. Although essential in the ability to polymerize host cell actin during early and late stages of infection for spotted fever group (SFG) *Rickettsia*, overall growth kinetics in cells were augmented compared to *R*. *parkeri* WT [23]. As multiple rickettsial molecules have recognized involvement in attachment to and invasion of host cells, compensation of rickettsial factors necessary for survival are likely present.

Due to the fast intermittent feeding biology of fleas, the initiation of bloodmeal digestion can occur as early as 6 hours post-feeding [55]. Additionally, the digestive process occurs within the midgut lumen and fleas are armed with the capacity to elicit an immune response to invading pathogens (reviewed in [40]). Thus, to avoid rapid excretion or detection by immune mechanisms, flea-borne rickettsiae must quickly attach to and invade midgut epithelial cells. However, the factors facilitating rickettsial colonization in fleas remains undefined. In its biological vector, the *R*. *felis sca1*::*tn* mutant’s growth was significantly reduced compared to *R*. *felis* WT, suggesting its inadequacy to colonize the flea at early stages of infection (*e*.*g*., host cell attachment or evasion of the flea’s immune response). It has been shown that fleas mount a transcriptional response against invading *Rickettsia typhi* during midgut infection [28,29] and *R*. *felis* during salivary gland infection [13]. However, the rickettsial-specific molecules involved in evading the arthropod’s natural immune response remain to be elucidated. Additionally, while protein expression of the 5 most prevalent Scas in *R*. *typhi* revealed differential expression between vertebrate and vector hosts; *R*. *typhi* Sca1 was not shown to be expressed during flea infection [56]. Differences in the observed phenotype of the current study may be due to *Rickettsia* sp. examined, *R*. *typhi* versus *R*. *felis*. More likely, the examination in the current study has identified a temporal necessity for *sca1* during flea infection. Temporal expression of pathogen determinants has been identified for other vector-borne pathogens, such as *Borrelia burgdorferi* and *Y*. *pestis* (reviewed in [19,57]). If Sca1 is implicated in the recognition of and adherence to host cells, then expression may be below the limit of detection at later stages of flea infection. Thus, a temporal assessment of rickettsial Sca expression profiles during flea infection is warranted to gain a thorough understanding of the factors essential for initial colonization, replication, and transmission.

Insect-borne typhus group *Rickettsia*, such as *R*. *typhi* and *Rickettsia prowazekii*, are known to colonize the insect’s midgut epithelium in which exponential growth causes host cell lysis, subsequently releasing rickettsiae back into the midgut lumen [58–60]. Extracellular rickettsiae are then excreted into insect feces, which is a primary mechanism of horizontal transmission to vertebrate hosts. The detection of *R*. *felis* WT in flea feces throughout the 28-day time course suggests a similar transmission mechanism [12]. In the current study, the inability of the *R*. *felis sca1*::*tn* mutant to be detected in flea feces at any time point may be a reflection of rickettsial load. Thus, if rickettsial loads within the flea correlate with detection within feces, a defect in *R*. *felis sca1*::*tn* may prevent the prerequisite steps essential for fecal transmission of insect-borne rickettsiae. With the current lack of appropriate vertebrate models to assess the contribution of *R*. *felis* transmission through feces, the implication of reduced rickettsial loads and this route of exposure requires further investigation.

Although *R*. *felis sca1*::*tn* had a reduced rickettsial load during flea infection compared to *R*. *felis* WT, it persisted within the flea cohort throughout the 28-day time course of this study, which is representative of the average adult flea lifespan. Detection of *R*. *felis* within salivary glands as early as 1 dpe is known [13]. Comparatively, *R*. *felis sca1*::*tn* was observed in flea salivary glands for at least 7 days, suggesting its ability to disseminate to other flea tissues was not fully impaired. Due to the inefficiency to induce *R*. *felis* systemic infection in mouse models, cofeeding bioassays were used to assess transmission of rickettsiae between vectors. While deposition of infectious salivary secretions into the skin of vertebrate hosts does not guarantee transmission of the agent that progresses to a systemic infection, detection of rickettsiae in the host skin at the arthropod feeding site is associated with transmission to proximal feeding arthropods [10,36]. Indeed, *R*. *felis sca1*::*tn* can be inoculated into murine skin and acquired by cofeeding, naïve fleas. Although *R*. *felis sca1*::*tn* was acquired orally during feeding in the artificial host, migrated to flea salivary glands, secreted during subsequent feeding events on vertebrate hosts, and acquired by neighboring fleas, a diminished transmission phenotype was observed compared to *R*. *felis* WT. Similar to the observations for fecal detection, reduced rickettsial loads in infected donor fleas likely influences transmission efficiency.

Recent advances in rickettsial genetics have provided several mutants of tick-borne rickettsiae. Yet, limited insect-associated rickettsial mutants have been developed [54,61,62]. To fully understand the complex biology of these obligate intracellular bacteria, the interplay of rickettsial factors during lifestyle changes between both mammalian and vector hosts must be examined. In the current study, transposon mutagenesis was employed to obtain randomized insertions with the *R*. *felis* genome, providing new resources to elucidate the function of rickettsial molecules involved in host infection. A clonal *R*. *felis* mutant with an insertion in the *sca1* gene was further examined to identify novel phenotypes associated with culture conditions and flea infection. Cell culture revealed an enhanced growth phenotype, yet dissemination was limited compared to the wild-type strain. Interestingly, a reduced rickettsial load observed in the flea vector exposed to *R*. *felis sca1*::*tn* correlated with decreased transmission potential. While several factors can contribute to the differences observed, the data suggest that *R*. *felis sca1* is associated with early infection of the vector and efficient transmission. Future studies utilizing complementation techniques and molecular reagents specific to *R*. *felis* Sca1 in the biological system presented within will facilitate full elucidation of Sca1’s role in transmission by the vector.

## Materials and methods

### Ethics statement

All animal research was performed under the approval of the University of South Alabama Institutional Animal Care and Use Committee (protocol number: 1489181).

### *Rickettsia* and cell lines

Rickettsial isolate, *R*. *felis* str. LSU passage 3, was maintained in an *Ixodes scapularis-*derived embryonic cell line (ISE6) using modified L15B medium [32]. Briefly, L15B medium was supplemented with 10% heat-inactivated fetal bovine serum (FBS), 5% tryptose phosphate broth, and 0.1% bovine lipoprotein to a final pH of 7.0–7.2. Rickettsiae were cultured until reaching 80–90% infectivity visualized by Diff-Quik staining as previously described [10,13,51].

### Rickettsial transformation

The *R*. *felis* str. LSU transposon mutants were generated using a modified pCis-mCherry-SS *Himar* A7 plasmid [32]. The plasmid carries sequences encoding a *mCherry* fluorescence marker and the *aadA* gene that confers resistance against spectinomycin and streptomycin with expression driven by the *Anaplasma marginale transcriptional regulator 1* (Am-Tr1) promoter [32]. The transposon is flanked by nine base pair inverted repeats recognizable by the *Himar1* transposase where 1,833 base pairs were inserted into the rickettsial genome. Rickettsial transformants were serially passed on ISE6 cells using L15B medium supplemented with 100μg/ml spectinomycin and streptomycin until clonal populations were achieved by limiting dilution method [33].

### Determination of *Himar1* insertion sites

To determine the *Himar1* insertion site, rickettsial stocks were sucrose purified by needle lysis as previously described [63]. Genomic DNA (gDNA) was extracted using the DNeasy Blood and Tissue Kit, according to the manufactures protocol (Qiagen). Prior to whole genome sequencing, the integrity of DNA fragments was visualized by agarose gel electrophoresis. The sequencing was carried out with an Ion Torrent Personal Genome Machine (PGMTM) System on a 316 chip. Sequences were aligned to the annotated *R*. *felis* URRWXCal2 genome as a reference database (NCBI GenBank accession number: CP000053.1). Insertions identified by genome sequencing were confirmed by PCR followed by Sanger sequencing using both a semi-random nested PCR method [37] and PCR-amplified products cloned into the pCR4-TOPO vector (Invitrogen) (**S1 Table**). Sequences were Sanger sequenced following Azenta Life Sciences specifications and aligned to the *R*. *felis* reference genome using the GenBank database using BLAST for further analyses by SnapGeneⓇ (Version 6.0.4) software. All whole genome sequences are deposited into the NCBI Sequence Read Archive under the BioProject accession PRJNA896619.

### Characterization of *R*. *felis sca1*::*tn*

To determine clonality of the *R*. *felis sca1*::*tn* mutant population, primers were designed to amplify flanking regions of the confirmed transposon insertion site (**S2 Table**). Amplicon specificity was validated by Sanger sequencing and aligned to the *R*. *felis* reference genome using NCBI BLAST. Bacterial populations were screened for clonality prior to each experiment. To determine alterations in *sca1* expression, primers were designed to amplify upstream and downstream of the known transposon insertion site (**S3 Table**). Semi-purified rickettsiae were harvested through needle-lysis followed by 2 μm filtration to remove large host cell debris and stored in TRIzol reagent (Invitrogen). Total RNA was isolated using Zymo mini-RNA kit, cleaned with the Zymo Clean and Concentrator kit, and any residual DNA was depleted using TurboDNase treatment (Ambion). cDNA was synthesized using iScript (Bio-rad) with random hexamers. A no reverse transcriptase control was used to confirm the absence of gDNA.

### *In vitro* infection assays

For analysis of rickettsial cell attachment and growth kinetics by immunofluorescence, ISE6 cells were seeded onto glass coverslips in 24-well plates at a density of 8x10^5^ cells/well or 8-well chamber slides at a density of 5x10^5^ cells/well and incubated at 32°C for 48 hours. To detect genomic equivalents during growth curve analysis, ISE6 cells were seeded in 48-well plates at a density of 5x10^5^ cells/well. Rickettsiae were enumerated by *Bac*Light viability stain kit [64] to determine a multiplicity of infection (MOI) of 10 rickettsiae/cell. Host cell contact was induced by centrifugation at 300 x g for 5 minutes and unbound bacteria were removed following specific incubation times described below. For the cell attachment assay, unbound rickettsiae were removed and infected cells were washed with PBS and fixed for immunofluorescence staining at 10-minute intervals for the first 30 minutes of infection. Microscopy images from 2 experiments with 10 fields of view were quantified.

For growth curve analysis, rickettsiae were added to ISE6 cells in tissue culture plates, centrifuged, and incubated at 32°C for 1 hour to allow rickettsiae to attach to host cells. At 1-hour post-infection (hpi), unbound rickettsiae were removed and samples were considered time point 0 where rickettsial growth was calculated as a change over time. The remaining wells were replaced with maintenance media for the duration of the experiment (7 days) where entire well volumes (both intracellular and extracellular rickettsiae) were collected every other day for rickettsial enumeration of genomic equivalents by qPCR (**S4 Table**). A total of 3 independent experiments were performed with 3 technical replicates per experiment for both *R*. *felis* WT and *R*. *felis sca1*::*tn*.

### *In vitro* immunofluorescence staining

Cells were fixed using 4% paraformaldehyde (PFA) for 20 minutes and washed thoroughly with PBS. To distinguish between cell attachment and invasion, extracellular bacteria were stained with rabbit anti-*Rickettsia* I1789 antibody (provided by Ted Hackstadt) followed by the secondary antibody, Alexa 594 goat anti-rabbit (Invitrogen, A11005; 1:1000 dilution). To subsequently stain all bacteria (intracellular and extracellular), cells were permeabilized and again stained with rabbit anti-*Rickettsia* I1789 antibody followed by the secondary antibody, Alexa 488 goat anti-rabbit (Invitrogen, A11008; 1:1000 dilution). Coverslips were mounted with VectaShield HardSet antifade mounting medium with DAPI (Vector Laboratories Inc.) for nuclear staining. Samples were visualized and quantified using a Nikon A1 microscope (S10RR027535).

For growth curves, cells were permeabilized using 0.5% Triton-X100 for 15 minutes and blocked with 3% BSA for 1 hour. Coverslips were then probed for rickettsiae using rabbit anti-*Rickettsia* I1789 antibody diluted 1:1000, followed by the secondary antibody, Alexa 488 goat anti-rabbit (Invitrogen, A11008; 1:1000 dilution). For all immunofluorescence assays, samples with secondary antibody only served as a control for non-specific binding of the Alexa fluor antibodies.

### Rickettsial isolation from blood

To re-isolate rickettsiae from prepared bloodmeals, 1 mL of *R*. *felis* WT- and *R*. *felis sca1*::*tn*-infected ISE6 cells were prepared as previously described [10]. Cell pellets were incubated in microcentrifuge tubes in an artificial dog unit for 48 hours to mimic flea infection temperature range. Host cells were then lysed, and cell debris was pelleted at 300 x g for 5 minutes. The supernatant was collected and filtrated through a 2 μm filter. Rickettsiae were enumerated by *Bac*Light viability stain kit [64] to determine a multiplicity of infection (MOI) of 50 bacteria/cell. Cells were infected in the same manner as growth curve analyses, in which whole well contents were collected every other day for 1 week. Similarly, rickettsial genome equivalents were calculated as a change over time by qPCR (**S4 Table**). A total of 2 experiments with 3 technical replicates/experiment were completed for both *R*. *felis* WT and *R*. *felis sca1*::*tn*.

### Flea infection

Cat fleas were purchased from Elward II Laboratory (Soquel, CA) and maintained using an artificial dog system [65]. Prior to use in each bioassay, a subset of fleas was confirmed to be *Rickettsia* free using qPCR protocols amplifying *R*. *felis ompB* gene [13]. For flea infections, cages were prepared with approximately 200 mixed-sex cat fleas and prefed heat-inactivated bovine blood (HemoStat Laboratories) for 24 hours. Following prefeeding, fleas were starved for 6 hours and exposed to a *R*. *felis* WT- or *R*. *felis sca1*::*tn*-infected bloodmeal at a low (1.5 x 10^10^ rickettsiae) or high dose (5 x 10^10^ rickettsiae) prepared as previously described [10,12]. Fleas were allowed continuous access to the infectious bloodmeal for 48 hours after which it was replaced with uninfected, defibrinated blood for the remainder of the study. A total of 20 fleas (10 male and 10 female) were collected weekly, surface sterilized [13], and homogenized using stainless steel beads in a TissueLyser II (Qiagen) prior to gDNA extraction and subsequent qPCR analysis with primers listed in **S4 Table** to determine rickettsial loads within individual fleas. To assess rickettsial loads per 1 mg of flea feces, feces were collected weekly [12] and subjected to gDNA extraction following the manufacturer’s instruction for blood isolation. A total of 3 independent replicates (60 fleas per time point) over a 28-day period were analyzed for both *R*. *felis* WT and *R*. *felis sca1*::*tn* mutant experimental groups.

### Flea microdissections, sections, and immunofluorescence staining

For salivary gland dissections, fleas were collected 48 hours post-exposure and 7 days post-exposure (dpe). Female fleas were surface sterilized, and microdissected in sterile phosphate-buffered saline (PBS) using a stereo microscope. Rinsed salivary glands were placed onto slides, fixed with 4% PFA and stored at 4°C until IFA staining was performed. For flea sections, whole fleas were submersed in 10% neutral buffered formalin for a minimum of 24 hours. Slides containing formalin-fixed paraffin-embedded flea sections (5 μm) were heated at 65°C for 15 minutes and deparaffinized by repeated immersions in Hemo-De (Electron Microscopy Sciences). Slides were rinsed with PBS, antigen retrieval, and immunofluorescence staining was performed as previously described [30, 31, 52]. Briefly, slides were incubated with mouse polyclonal antisera to *R*. *felis* [52, 64] at a dilution of 1:100 for 1 hour and subsequently incubated with Alexa 488 goat anti-mouse (Invitrogen, A11001; 1:1000 dilution). Fleas were counterstained with 0.1% Evans blue in PBS at 37°C for 30 minutes and mounted with VectaShield HardSet antifade mounting medium containing DAPI (Vector Laboratories Inc.) for nuclear staining. Samples were visualized using a Nikon A1 microscope (S10RR027535).

### Flea cofeeding

Five-week-old, male, C3H/HeJ mice (Jackson Laboratory) were used as a murine model for transmission studies. For cofeeding bioassays, donor fleas were exposed to either *R*. *felis* WT- or *R*. *felis sca1*::*tn*-infected bloodmeals, independently, for 48 hours at an infectious dose of 5 x 10^10^ rickettsiae. In parallel, recipient (naïve) fleas were exposed to a bloodmeal supplemented with the fluorescent biomarker, rhodamine B (RhoB) at a working concentration of 0.025%, for 48 hours [10, 31] (**Fig 8**). Five days post-exposure to the infectious bloodmeal, 10 donor and 10 recipient mixed-sex fleas were combined into feeding capsules made from modified 1.7 ml microcentrifuge tubes [10]. Capsules were attached to the shaved flank of mice and fleas were allowed to feed for three 12-hour increments. Fleas and skin biopsies at the site of flea feeding were collected after the final feeding time point (7 dpe). Fleas were prepared for qPCR analyses as described for the infection bioassay. A total of 3 mice were used per experimental group, along with a control mouse exposed to uninfected fleas only. Rickettsiae were not detected in control samples by qPCR.

### DNA extraction and qPCR

To determine genome equivalents of rickettsiae, gDNA was extracted using the DNeasy Blood and Tissue Kit (Qiagen) following the manufacturer’s instruction. Rickettsial and host gene copies were quantified by qPCR with the appropriate primers and probes (**S4 Table**) using iTaq Universal Probes Supermix (Bio-Rad) on a LightCycler 480 II (Roche Life Sciences). Standard curves were generated by creating 10-fold serial dilutions of pCR4-TOPO plasmids containing the *R*. *felis ompB*, *C*. *felis 18sRNA*, or *ISE6 calreticulin* genes to quantify each target sequence. Amplification conditions were as follows: an initial denaturation step at 95°C for 3 minutes, followed by 45 cycles of denaturation at 95°C for 15 seconds, annealing and elongation at 60°C for 60 seconds with fluorescence acquisition in single mode.

### Statistical analyses

To compare growth kinetics and cell association of *R*. *felis sca1*::*tn* to *R*. *felis* WT in ISE6 cells, a two-tailed t-test was performed to determine differences between the means at a given time point. A two-way analysis of variance (ANOVA) was performed for flea infections to compare differences in variance between *R*. *felis sca1*::*tn* and *R*. *felis* WT overtime. During the cofeeding bioassay, a Fisher’s exact test for significance was used to examine independence between the proportion of *R*. *felis*-infected recipient fleas for each rickettsial strain. To determine significant differences between flea donor loads, a Mann Whitney test was performed. A p value ≤ 0.05 was considered statistically significant. All statistical analyses were performed using Prism 8 software (GraphPad Software). Data used for figures are displayed in **S1 Data**.

## Supporting information

S1 Table Primers for Sanger sequencing. (DOCX)

S2 Table Primers for examining clonality. (DOCX)

S3 Table Primers for RT-PCR. (DOCX)

S4 Table Primers and probes for qPCR. (DOCX)

S1 Data Excel sheets providing numerical data used for compilation of figures and statistical analyses. (XLSX)

S1 Fig Polar effects of Himar1 on adjacent genes in R. felis sca1::tn. A) Graphical representation of the genes downstream of *sca1* with primers sets used for RT-PCR indicated by blue arrows. B) Representative agarose gel of the amplification of adjacent genes downstream of *sca1* using PCR from cDNA samples of *R*. *felis sca1*::*tn* (lanes 2, 6, 10) and *R*. *felis* WT (lanes 1, 5, 9). Rickettsial DNA samples were used as controls for gene amplification (lanes 13–18). cDNA samples lacking reverse transcriptase were used as a negative control (lanes 3, 4, 7, 8, 11, 12).
(TIF)

S2 Fig Re-isolation of rickettsiae from blood. Rickettsiae were lysed from host cells and ISE6 cells were infected with semi-purified *R*. *felis* WT or *R*. *felis sca1*::*tn* after a 48-hour incubation period in bovine blood. Growth curve is measuring rickettsial genome equivalents by qPCR. Data are representative of mean ± SEM from two experiments, with 3 technical replicates, and normalized to input bacteria at 1 hpi. Significance was assessed at a 95% confidence interval by unpaired t-test to assess variation in the means from wild-type at a given time.
(PDF)
