# Supplementary material for: Transposon mutagenesis of Rickettsia felis sca1 confers a distinct phenotype during flea infection
Source: PLoS Pathog. 2022 Dec 21;18(12):e1011045. doi: 10.1371/journal.ppat.1011045 (PMC9815595; doi:10.1371/journal.ppat.1011045)
Supplement: S1 Table — (DOCX) [file ppat.1011045.s001.docx]

**S1 Table.**

**Primers for Sanger sequencing.**

| **Oligo name**: primer set  (5' - 3') | **Sequence** | **Citation** |
| --- | --- | --- |
| Univ.primer1 | GCTAGCGGCCGCACTAGTCGANNNNNNNNNNCTTCT | [1] |
| mCherry.EXT | TTCGCCTTCGCCTTCGATTTCAAACT | This study |
| Univ.primer2 | GCTAGCGGCCGCACTAGTCGA | [1] |
| mCherry.INT | TCCATGTGCACCTTAAATCTCATAAACTCTTT | This study |
| mCherry.UP.  OUT | ATTATCTTCCTCTCCCTTGCTGACC | [2] |
| A1.FOR | TCATACATAATGTTAATGCAACAGT | This study |
| A1.REV | GACATTGCTTGCCTTTATAACAT |  |
| A2.FOR | TCATACATAATGTTAATGCAACAGT | This study |
| A2.REV | CGACAATAATATTCTAGGTATAGCTT |  |
| A4.FOR | TCATACATAATGTTAATGCAACAGT | This study |
| A4.REV | GATGTATTGGTATGGAAGAG |  |
| A5.FOR | TCATACATAATGTTAATGCAACAGT | This study |
| A5.REV | ATTCATTAGCTTCGTGAATCGT |  |
| B2.FOR | TCATACATAATGTTAATGCAACAGT | This study |
| B2.REV | GATATTATCGCATCCCCAACTTG |  |
| C1ii.FOR | TCATACATAATGTTAATGCAACAGT | This study |
| C1ii.REV | CTTCATCAGAGAGTATAAATTCTAGTGAC |  |
| C4.FOR | CAGCCCGTCATACTTGAAGCTAGGC | This study |
| C4.REV | CTCTTCATTGCTTACTTCTACCTTATC |  |
| D6.FOR | TCATACATAATGTTAATGCAACAGT | This study |
| D6.REV | CTTTGCCGTATCCAAGTCTATA |  |

**References**

1. Lamason RL, Bastounis E, Kafai NM, Serrano R, Del Alamo JC, Theriot JA, et al. *Rickettsia* Sca4 reduces vinculin-mediated intercellular tension to promote spread. Cell. 2016;167(3):670-83.e10.
2. Lynn GE, Burkhardt NY, Felsheim RF, Nelson CM, Oliver JD, Kurtti TJ, et al. *Ehrlichia* isolate from a Minnesota tick: characterization and genetic transformation. Appl Environ Microbiol. 2019;85(14).
