# Supplementary material for: Transposon mutagenesis of Rickettsia felis sca1 confers a distinct phenotype during flea infection
Source: PLoS Pathog. 2022 Dec 21;18(12):e1011045. doi: 10.1371/journal.ppat.1011045 (PMC9815595; doi:10.1371/journal.ppat.1011045)
Supplement: S2 Table — (DOCX) [file ppat.1011045.s002.docx]

**S2 Table.**

**Primers for examining clonality.**

| **Oligo name**: primer set (5' - 3') | **Sequence** | **Citation** |
| --- | --- | --- |
| A1.clonality.FOR | GAGATAGCATTATTCAGTCGGAAAATT | This study |
| A1.clonality.REV | GACATTGCTTGCCTTTATAACAT |  |
| A2.clonality.FOR | TGGATGCGTGGTATGTACGG | This study |
| A2.clonality.REV | CCTCGGTAATTCTTTTTGTCCG |  |
| A4.clonality.FOR | GCACGTAATATATGTTCTTTCATATTCCT | This study |
| A4.clonality.REV | GATGTATTGGTATGGAAGAG |  |
| A5.clonality.FOR | GCCTCAATATATCGCTATCTTGC | This study |
| A5.clonality.REV | CAGCCCGAAGATTTGATCTT |  |
| B2.clonality.FOR | TGCCGCAATAATTGGTACTAC | This study |
| B2.clonality.REV | GATATTATCGCATCCCCAACTTG |  |
| C1ii.clonality.FOR | CTTCATCAGAGAGTATAAATTCTAGTGAC | This study |
| C1ii.clonality.REV | GCGTTACTTTAGATTCTAGTAATTGTG |  |
| C4.clonality.FOR | CGGCTTACCTGACGAAATAGAA | This study |
| C4.clonality.REV | CTCTTCATTGCTTACTTCTACCTTATC |  |
| D6.clonality.FOR | GTCCTTATCATTATAACGATCAGTTGG | This study |
| D6.clonality.REV | CTTTGCCGTATCCAAGTCTATA |  |
