# Supplementary material for: Transposon mutagenesis of Rickettsia felis sca1 confers a distinct phenotype during flea infection
Source: PLoS Pathog. 2022 Dec 21;18(12):e1011045. doi: 10.1371/journal.ppat.1011045 (PMC9815595; doi:10.1371/journal.ppat.1011045)
Supplement: S3 Table — (DOCX) [file ppat.1011045.s003.docx]

**S3 Table.**

**Primers for RT-PCR.**

| **Oligo name**: primer set  (5' - 3') | **Sequence** | **Citation** |
| --- | --- | --- |
| sca1.UP.FOR | TGGATGCGTGGTATGTACGG | This study |
| sca1.UP.REV | GTCGATTTCAACGTCAAACCCA |  |
| sca1.DWN.FOR | GAAGTCACGAGACGGGGT | This study |
| sca1.DWN.REV | CTTAACACTACCTTGATGGCTTCTAT |  |
| RF_0023.FOR | CAATCTACATTTAACGCTTCCGC | This study |
| RF_0023.REV | ATTAGGGTGATGCTTATAGTTTTGATTATTT |  |
| RF_0024.FOR | CAGAAGCAAAGGCAATCACATTC | This study |
| RF_0024.REV | CAACTGTGCTAGATACCAATCACT |  |
| RF_0025.FOR | AGTGAAAAACGATTTGTAACAGTCAG | This study |
| RF_0025.REV | AGCCTATAAGGTGATACAGCATCT |  |
