# Supplementary material for: Transposon mutagenesis of Rickettsia felis sca1 confers a distinct phenotype during flea infection
Source: PLoS Pathog. 2022 Dec 21;18(12):e1011045. doi: 10.1371/journal.ppat.1011045 (PMC9815595; doi:10.1371/journal.ppat.1011045)
Supplement: S4 Table — (DOCX) [file ppat.1011045.s004.docx]

**S4 Table.**

**Primers and probes for qPCR.**

| **Oligo name**:  primer set (5' - 3') | **Sequence** | **Citation** |
| --- | --- | --- |
| Rfel.OmpB.FOR | TAATTTTAACGGAACAGACGGT | [1] |
| Rfel.OmpB.REV | GCCTAAACTTCCTGTAACATTAAAG |  |
| Rfel.OmpB.HEX/FAM | HEX/ or FAM/TGCTGCTGGTGGCGGTGC |  |
| 18srRNA.FOR | GAGTTCCGACCAGAGATGGA | This study |
| 18srRNA.REV | CGCAGAAACTACCATCGACA |  |
| 18srRNA.FAM | FAM/TGCCTTGCTCACCGTTTGACTTGGTG |  |
| Ise6.cal.FOR | AGCAGGGAACTTTCAAGCTG | [2] |
| Ise6.cal.REV | AGAAAGGCTCGAACTTGGTG |  |
| Ise6.cal.HEX | HEX/AGACCTCTGAAGATGCCCGCTTT |  |

**References**

1. Odhiambo AM, Maina AN, Taylor ML, Jiang J, Richards AL. Development and validation of a quantitative real-time polymerase chain reaction assay specific for the detection of *Rickettsia felis* and not *Rickettsia felis*-like organisms. Vector Borne Zoonotic Dis (Larchmont, NY). 2014;14(7):476-81.
2. Harris EK, Jirakanwisal K, Verhoeve VI, Fongsaran C, Suwanbongkot C, Welch MD, et al. The role of Sca2 and RickA in the dissemination of *Rickettsia parkeri* in *Amblyomma maculatum*. Infect Immun. 2018;86.
