# Supplementary figures and images for: Transposon mutagenesis of Rickettsia felis sca1 confers a distinct phenotype during flea infection
Source: PLoS Pathog. 2022 Dec 21;18(12):e1011045. doi: 10.1371/journal.ppat.1011045 (PMC9815595; doi:10.1371/journal.ppat.1011045)

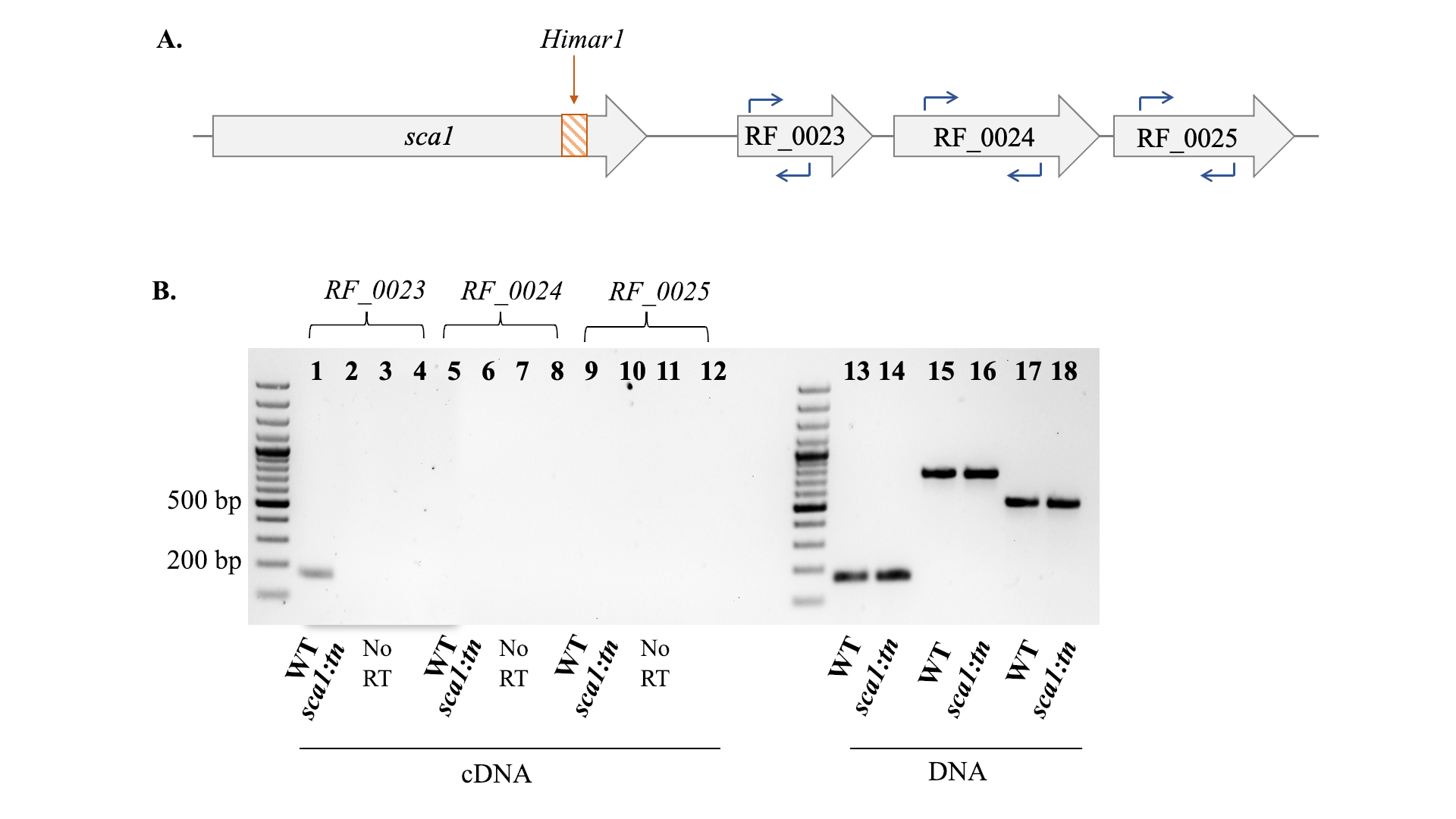

Supplement: S1 Fig — A) Graphical representation of the genes downstream of sca1 with primers sets used for RT-PCR indicated by blue arrows. B) Representative agarose gel of the amplification of adjacent genes downstream of sca1 using PCR from cDNA samples of R. felis sca1::tn (lanes 2, 6, 10) and R. felis WT (lanes 1, 5, 9). Rickettsial DNA samples were used as controls for gene amplification (lanes 13–18). cDNA samples lacking reverse transcriptase were used as a negative control (lanes 3, 4, 7, 8, 11, 12). (TIF) [file ppat.1011045.s006.tif]

## Isolation from blood

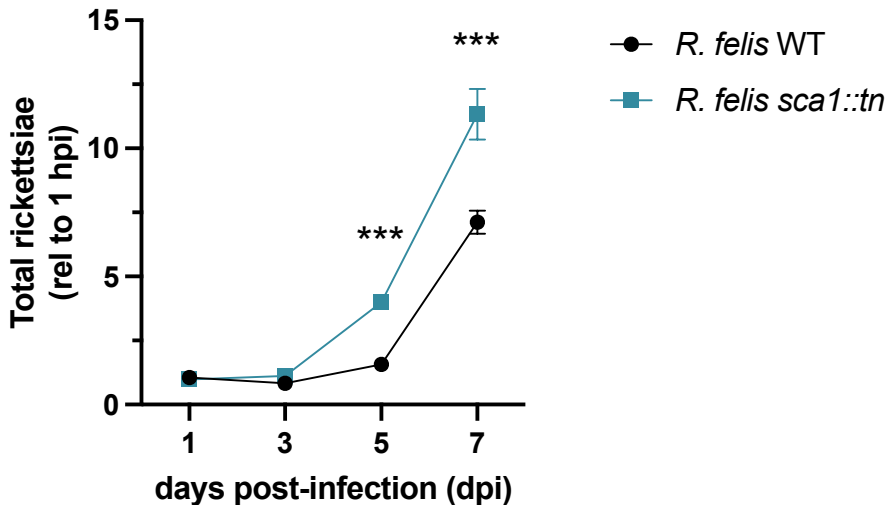

Supplement: S2 Fig — Rickettsiae were lysed from host cells and ISE6 cells were infected with semi-purified R. felis WT or R. felis sca1::tn after a 48-hour incubation period in bovine blood. Growth curve is measuring rickettsial genome equivalents by qPCR. Data are representative of mean ± SEM from two experiments, with 3 technical replicates, and normalized to input bacteria at 1 hpi. Significance was assessed at a 95% confidence interval by unpaired t-test to assess variation in the means from wild-type at a given time. (PDF) [file ppat.1011045.s007.pdf]
